# Photocycle alteration and increased enzymatic activity in genetically modified photoactivated adenylate cyclase OaPAC

**DOI:** 10.1016/j.jbc.2023.105056

**Published:** 2023-07-17

**Authors:** Katalin Raics, Katalin Pirisi, Bo Zhuang, Zsuzsanna Fekete, Nikolett Kis-Bicskei, Ildiko Pecsi, Kinga Pozsonyi Ujfalusi, Elek Telek, Yin Li, Jinnette Tolentino Collado, Peter J. Tonge, Stephen R. Meech, Marten H. Vos, Emoke Bodis, Andras Lukacs

**Affiliations:** 1 Department of Biophysics, Medical School, University of Pecs, Pecs, Hungary; 2 Laboratoire d’Optique et Biosciences, Ecole Polytechnique, Palaiseau, France; 3 Department of Physics, School of Physics and Materials Science, Nanchang University, Nanchang City, China; 4 Department of Chemistry, Stony Brook University, New York, USA; 5 School of Chemistry, University of East Anglia, Norwich, UK

**Keywords:** photoactivated adenylate cyclase, cAMP, proton-coupled electron transfer, PCET, transient absorption, transient fluorescence, BLUF domain, flavin, photochemistry

## Abstract

Photoactivated adenylate cyclases (PACs) are light activated enzymes that combine blue light sensing capacity with the ability to convert ATP to cAMP and pyrophosphate (PPi) in a light-dependent manner. In most of the known PACs blue light regulation is provided by a blue light sensing domain using flavin which undergoes a structural reorganization after blue-light absorption. This minor structural change then is translated toward the C-terminal of the protein, inducing a larger conformational change that results in the ATP conversion to cAMP. As cAMP is a key second messenger in numerous signal transduction pathways regulating various cellular functions, PACs are of great interest in optogenetic studies. The optimal optogenetic device must be “silent” in the dark and highly responsive upon light illumination. PAC from *Oscillatoria acuminata* is a very good candidate as its basal activity is very small in the dark and the conversion rates increase 20-fold upon light illumination. We studied the effect of replacing D67 to N, in the blue light using flavin domain. This mutation was found to accelerate the primary electron transfer process in the photosensing domain of the protein, as has been predicted. Furthermore, it resulted in a longer lived signaling state, which was formed with a lower quantum yield. Our studies show that the overall effects of the D67N mutation lead to a slightly higher conversion of ATP to cAMP, which points in the direction that by fine tuning the kinetic properties more responsive PACs and optogenetic devices can be generated.

The photoactivated adenylate cyclase (PAC) from the cyanobacterium *Oscillatoria acuminata* (OaPAC) is a recently discovered flavoprotein that translates a blue-light signal into the production of cAMP (1). OaPAC is a homodimer of a 366-aa protein comprising an N-terminal blue light using flavin (BLUF) (a blue-light using flavin adenine dinucleotide [FAD]) domain and a C-terminal class III adenylyl cyclase (AC) domain. The AC activity of OaPAC is stimulated by light up to 20-fold above basal levels in the dark (1).

Blue-light regulation of the majority of the PAC proteins is achieved by a BLUF domain. BLUF domains act as light sensing modules and are involved in a large range of light-controlled biological processes like bacteriochlorophyll biosynthesis, biofilm formation, phototaxis, and controlling levels of cyclic-AMP (2, 3, 4, 5, 6, 7, 8). Despite the diversity in the function of BLUF domains, the photosensing mechanism is similar— blue-light absorption by the FAD chromophore leads to a rearrangement of the hydrogen bonding network, which is reflected in a red-shift of ∼ 10 nm of the S_0_–S_1_ flavin transition (8).

The photoinduced mechanism of the BLUF domain is driven by the photochemistry of FAD—after blue light excitation, the flavin attracts an electron from the neighboring electron-rich amino acids like tryptophans or tyrosines (9, 10, 11). After the electron transfer step, either the anionic (FAD^•−^) or the neutral (FADH^•^) flavin radical is formed depending on the flavin environment. A similar process is crucial in the function of cryptochromes, where excitation of the oxidized flavin leads to electron transfer from the neighboring tryptophan, forming the anionic flavin radical followed by protonation (on the microsecond timescale) to yield a semiquinone flavin radical form (12, 13, 14, 15, 16). However, the actual role of electron transfer in BLUF domain proteins is still under debate, as it was not observed in activation of photopigment and PUC A protein (AppA) (17), BlsA (18), or BlrB (7), but was found to be crucial in PixD (5, 19) and PapB (20).

In the OaPAC BLUF domain (Fig. 1) a concerted proton-coupled electron transfer (PCET) process takes place: upon excitation of FAD an electron is transferred from a nearby tyrosine (Y6) to the flavin while simultaneously a proton is transferred from Y6 to the adjacent glutamine (Q48) and later to the flavin. Aside the involvement in the PCET process Q48 plays a central role in the photoactivation and the function of the protein as it is thought to tautomerize after the light excitation of flavin. Tautomerization of the glutamine was first proposed for AppA (21, 22, 23, 24, 25, 26, 27, 28) but later was proven to be present in PixD (29, 30, 31) as well. Based on these findings, it is a plausible assumption that during the PCET process Q48 tautomerizes, and this step is expected to be crucial in transmitting the signal to the C-terminal part of the protein where ATP is converted to cAMP. The role of Q48 should be further studied as it is expected to play a central role in the photoactivation and the overall function of OaPAC.

**Figure 1:**
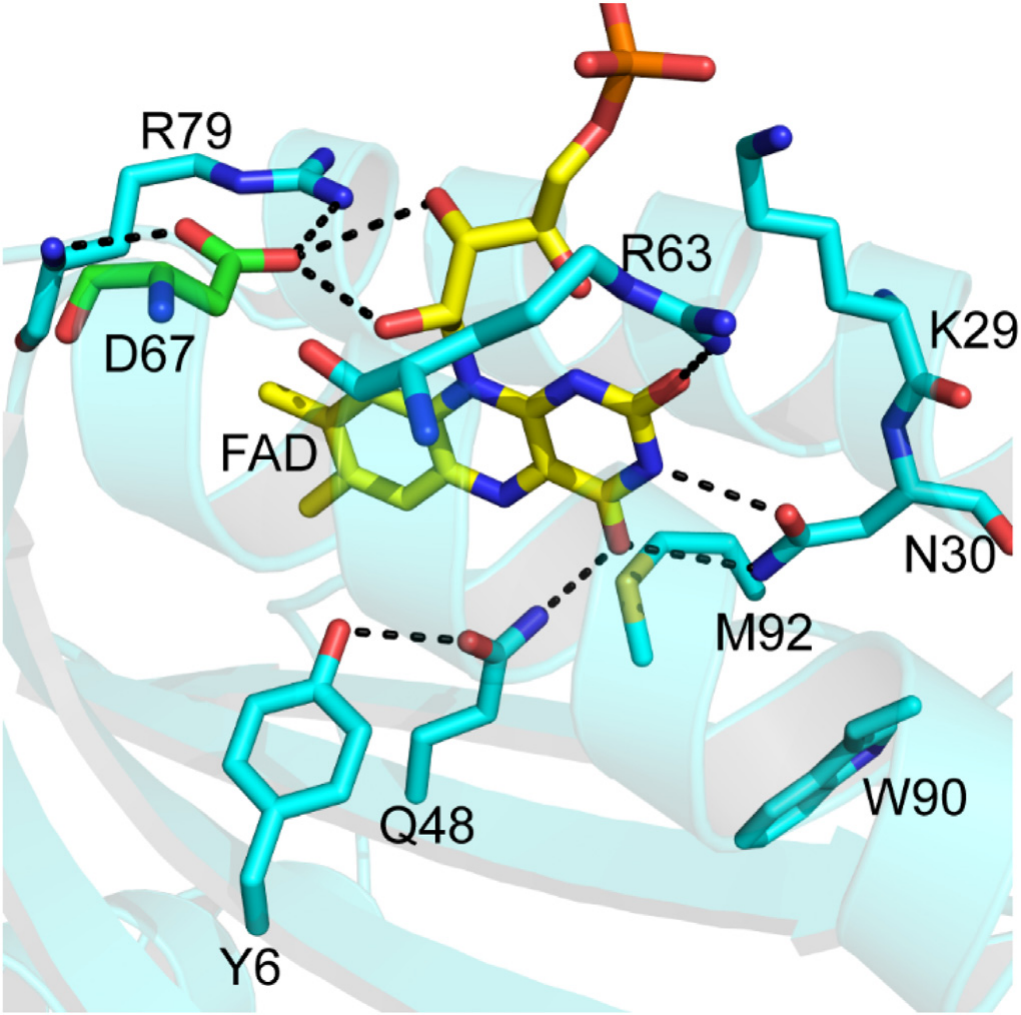
**Environment of FAD in OaPAC including the Y6, D67, Q48, and W90 amino acids, involved in the photophysics of OaPAC (PDB:****4yus****).** Y6 is the primary electron donor, but W90 can donate an electron as well. Q48 is crucial in the propagation of the signal from the BLUF domain toward the AC domain. D67 is in hydrogen bonding distance to R79 (∼3 Å) residue and to the ribityl chain (∼3.2 Å) of flavin. D67 is a hydrogen bond acceptor and mutating D67 to N, a neutral amino acid, modulates the electrostatic potential of flavin by altering the hydrogen boding network. AC, adenylate cyclase; BLUF, blue light using flavin; FAD, flavin adenine dinucleotide; OaPAC, photoactivated adenylate cyclase from *Oscillatoria acuminata*; PDB, Protein Data Bank.

PACs are important for applications in the life sciences as the light-controlled cAMP production is a promising optogenetic tool: EuPAC was expressed in the neurons of the marine gastropod *Aplysia* enabling photocontrol of neuron stimulation (32), and bPAC was utilized in transgenic mice in light-controlled flagellar beat of sperms (33). The latter has a 300-fold increase in cAMP conversion activity compared to OaPAC which has around a 20-fold activity increase (1, 34). It is therefore important to enhance control of the cyclase activity of OaPAC. In this work, we investigated the functional dynamics of D67N mutant OaPAC, which shows an ∼1.5-fold increase in light-induced AC activity of OaPAC as well as an acceleration of the forward and backward electron transfer processes and a decrease of the dark state recovery rate in the BLUF domain. The mutation slightly elevated the activity of the enzyme in the dark-adapted state as well, which points to a structural change extending to the enzymatic domain.

## Results and discussion

### Transient absorption measurements

Previously, ultrafast transient infrared and visible absorption spectroscopy were employed to investigate the photochemistry of OaPAC and it was established that after absorption of blue light by the flavin, a PCET takes place (35). During the PCET process, the primary electron donor is the conserved Y6 tyrosine (35). Photoexcitation of the flavin leads to the extraction of an electron from the tyrosine accompanied by the transfer of a proton from the same tyrosine resulting in the formation of neutral (36)tyrosine and flavin radicals. In our previous work, we demonstrated that the AC activity of OaPAC is linked to this PCET process: as the pK_a_ of the crucial tyrosine (Y6) was lowered from 9.9 to <7.7, the photocycle was halted at FAD^•−^ and no enzymatic activity was observed (35).

Adiabatic quantum mechanical/molecular mechanical simulations performed on Slr1694 (also called PixD), another intensively studied (5, 19, 37) BLUF domain protein, suggested that the replacement of the negatively charged aspartic acid D69 (D67 in OaPAC, Fig. 1) with a neutral or a positively charged residue will dramatically affect the electron transfer process (38). It was predicted that such mutations may demonstrate vastly different photocycle kinetics (38). Thus, here we investigate experimentally the potential effect of an analogous mutation on the photoactivation mechanism of OaPAC and measure the resulting enzymatic activity of the AC domain.

The primary photochemistry of the D67N mutant was characterized using transient absorption (TA) measurements. Ultrafast TA spectroscopy is a powerful method to characterize the electron transfer processes in flavoproteins (4, 5, 7, 10, 14, 39, 40, 41, 42, 43). Depending on the protein environment the flavin chromophore can exist in five different redox states (44), and these redox states possess distinct absorption spectra (Fig. S1*A*). Using these spectra one can perform spectral modeling in order to identify the flavin and amino acid radical species detected at different time delays after excitation (10, 14, 45, 46).

**Figure 2:**
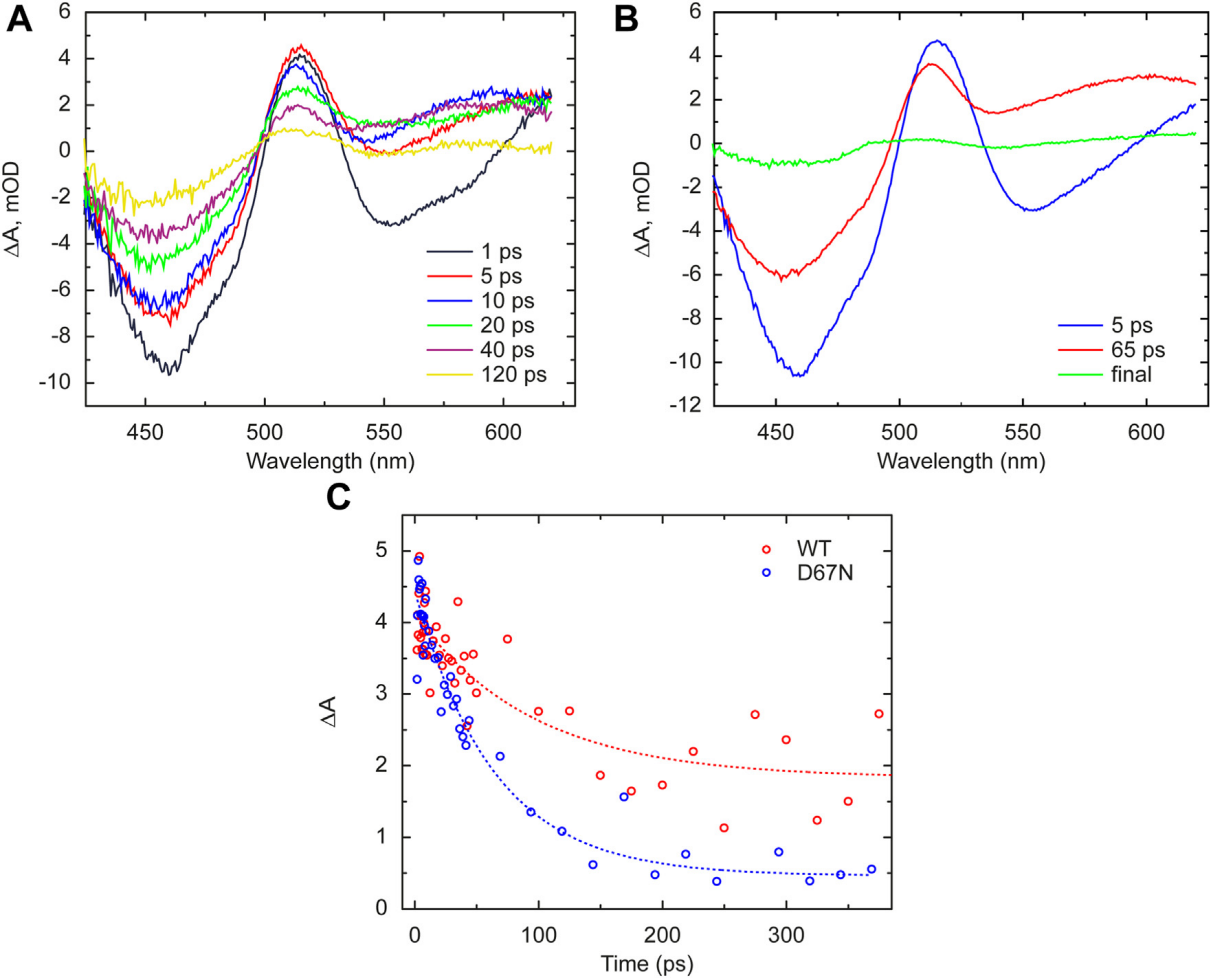
**Ultrafast transient absorption spectroscopy measurements.***A*, transient absorption measurements on the D67N mutant at indicated time delays *B*, EAS spectra obtained after global analysis. The time constants show that the excited state of FAD, formed right after excitation, decays in 5 ps to the next state which shows the presence of the neutral flavin radical. The final state is dominated by the signaling state and exist longer than the window of the measurement *C*, individual kinetics of the WT and D67N OaPAC measured at 505 nm. Applying a monoexponential fit the time constants for the WT and D67N were 90 ± 30 ps and 64 ± 8 ps, respectively. EAS, evolutionary-associated spectra; FAD, flavin adenine dinucleotide; OaPAC, photoactivated adenylate cyclase from *Oscillatoria acuminata*.

The TA spectra of D67N OaPAC measured at early time delays (Fig. 2*A*) are similar to those of other (oxidized) flavoproteins (10, 22), including WT OaPAC (35): an intense negative peak (bleach) is observed around 450 nm, which reflects disappearance of the S0–>S1 absorption of the flavin. The positive peak around 510 nm is attributed to the absorption of the excited state, whereas the negative broad peak ∼ 550 nm observed at the early time delays can be assigned to the stimulated emission of the flavin. The TA data for the WT protein could be globally fitted with three different time constants (35), 5 ps, 83 ps, and an infinite value (also called final state). Analysis of the corresponding evolutionary-associated spectra (EAS) indicated that the 5-ps component reflects FAD∗ decay. The 83-ps components reflects decay of both FAD∗ and formation of FADH^•^. The FAD∗ decay kinetics, by electron and proton transfer to the flavin, are thus dispersive (see fluorescence kinetics below) and the slower phases cannot be kinetically disentangled from that of the formation and decay of the FADH^•^ intermediate state. The final state represents the red-shifted FADox signaling state. Similarly to the WT measurements, the TA data of D67N was also globally fitted with three different time constants (Fig. 2*B*) of 5 ps, 65 ps, and infinite. The 5-ps EAS ascribed to FAD∗ decay resembles the 5 ps EAS of WT and EAS2 (65 ps phase for D67N) was equally modeled as a combination of the spectra of the excited flavin and the neutral flavin radical (Fig. S1*B*). The 5 ps component thus reflects the formation of the neutral flavin radical and the 65 ps component reflects the relaxation of the radical state (Fig. S2). Yet, the relative contribution of FAD∗ to EAS2 appears smaller for D67N (∼75% in WT and ∼60% in D67N), indicating also dispersive but faster overall FAD∗ decay (see also below). In addition, the second time constant is somewhat shorter in the mutant (65 ps *versus* 85 ps) which also suggests a faster recombination of the radical pair. In WT OaPAC (35) the ∼ 510 nm peak shifts toward 480 nm in ∼80 ps; this shift is due to the formation of the signaling state. A similar shift is observed in D67N OaPAC mutant, but the amplitude of the final state EAS is smaller in the mutant compared to WT (Fig. S3). Along with the finding that the spectrum of the signaling state is similar in WT and D67N OaPAC (Fig. 5*A* below), this indicates lower quantum yield of formation of the signaling state. Based on the amplitude ratios of the final state and the initially formed FAD∗ difference spectra (EAS final and 5-ps in Fig. S3) we estimate that the signaling state QY is ∼4-fold lower in D67N than in WT OaPAC.

**Figure 3:**
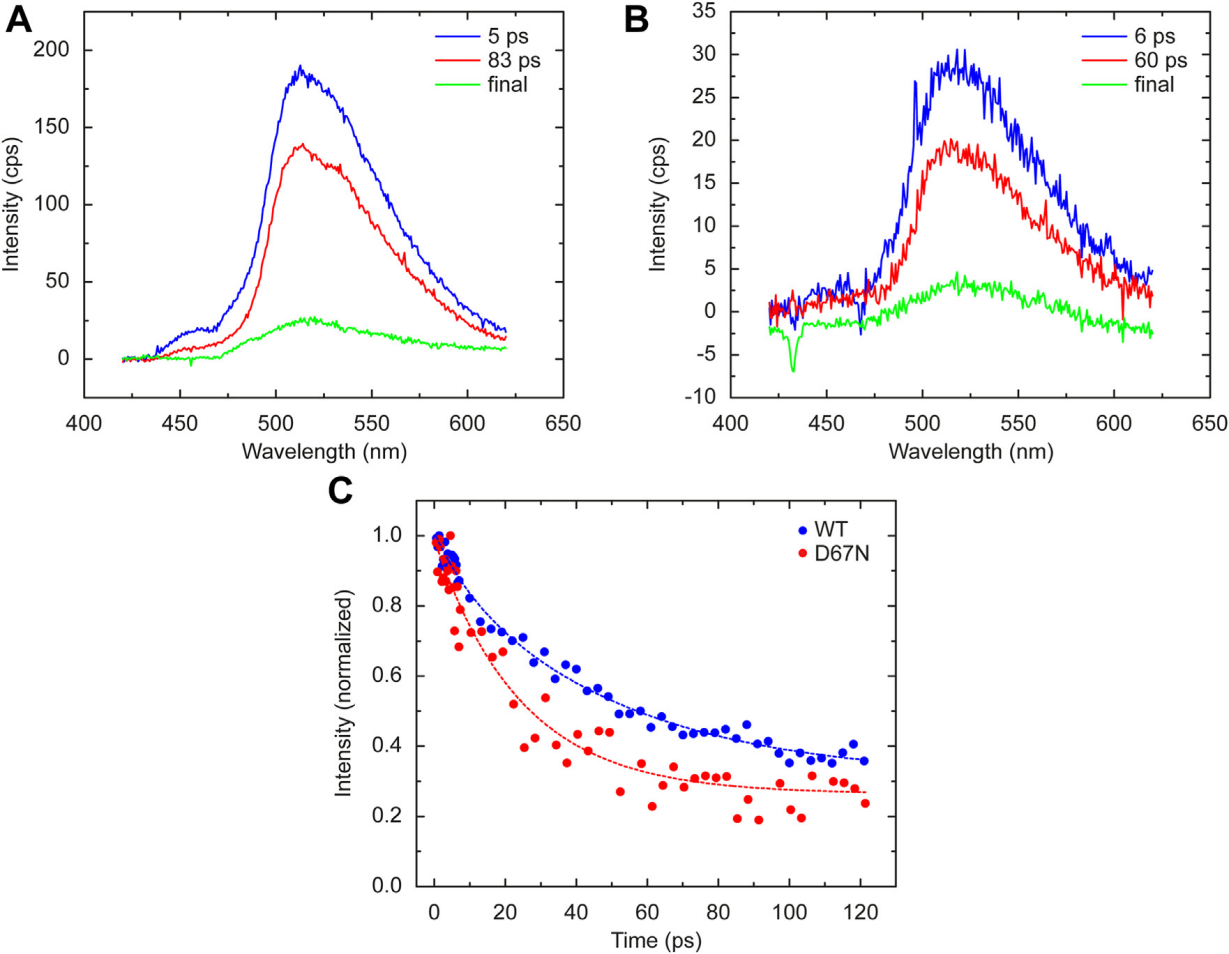
**Ultrafast transient fluorescence spectroscopy measurements.***A*, EAS spectra obtained by global analysis of the transient fluorescence data of WT. The result of the global fit shows a heterogenous decay of the flavin. *B*, EAS spectra obtained by global analysis of the transient fluorescence data of the D67N mutant. *C*, individual kinetics of the fluorescence decay observed at 513 nm. Using a monoexponential fit, we obtained a fluorescence lifetime of 40 ± 2 ps for WT and 25 ± 2 ps for D67N. EAS, evolutionary-associated spectra.

**Figure 4:**
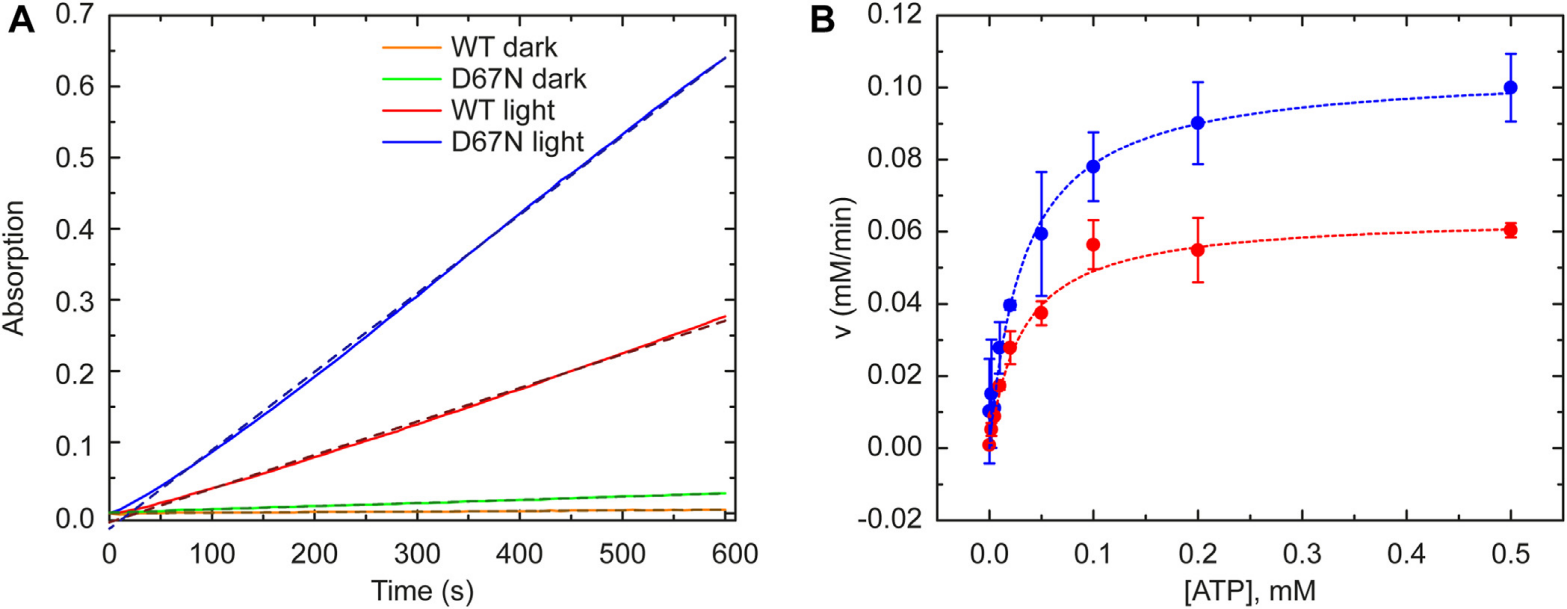
**Enzymatic activity of WT and D67N OaPAC.***A*, the kinetics of ATP conversion in the dark- and light-adapted state of WT and D67N OaPAC. In the dark, the ATP conversion rate is slightly higher in the D67N mutant (*green*) than in WT (*orange*). In the light-adapted state at 0.5 mM ATP the speed of the conversion was higher in the case of the mutant (*blue*) than in WT (*red*). *B*, Michaelis-Menten plot of the enzymatic activity of WT (*red*) and D67N (*blue*) under irradiation, *v*_max_ is increased in the D67N mutant. OaPAC, photoactivated adenylate cyclase from *Oscillatoria acuminata*.

**Figure 5:**
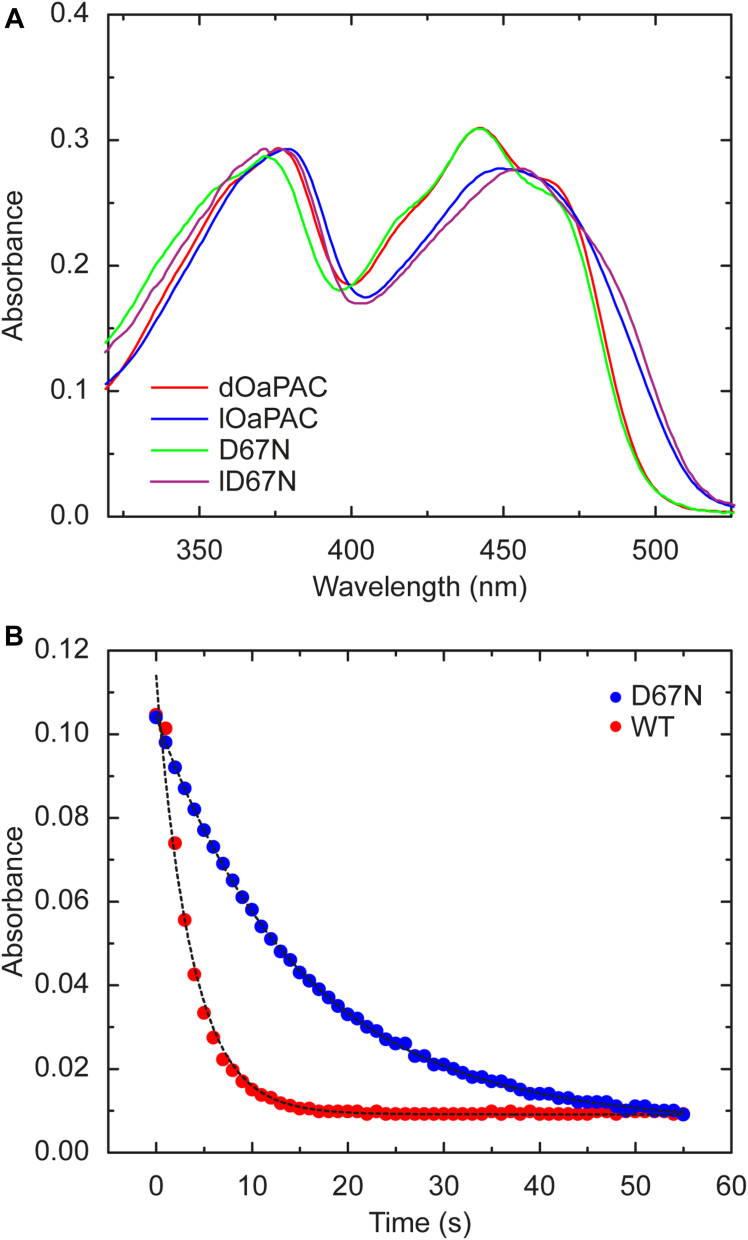
**Dark state recovery of WT and D67N OaPAC.***A*, absorption spectra of WT and D67N OaPAC in dark and light state. The absorption spectra of WT and the mutant are very similar in the dark adapted state: the peak of the S_0_–S_1_ transition is the same for both proteins with a maximum at 442 nm; a 4 nm blue shift of the S_0_–S_2_ transition is observed for the mutant (376 nm for the WT and 372 nm for the mutant). In light-adapted state the S_0_–S_1_ peak shifts to 452 nm in WT and to 456 nm in the mutant. The S_0_–S_2_ peak shifts from 376 nm to 379 nm in the WT, and from 372 nm to 376 nm in the mutant. *B*, recovery kinetics of WT (*red*) and D67N (*blue*) OaPAC, measured at 490 nm The time constant of dark state recovery is 3.6 s for WT and 15.3 s for D67N. OaPAC, photoactivated adenylate cyclase from *Oscillatoria acuminata*.

Observing the kinetics of the excited state absorption peak of the flavin (∼505 nm) the mutant’s relaxation appears slightly faster compared to WT (Fig. 2*C*); applying a monoexponential fit the time constants for the WT and D67N were 90 ± 30 ps and 64 ± 8 ps, respectively. This is due to faster FAD∗ decay kinetics (see below) as well as to the lower asymptotic value at this wavelength due to lower signaling state formation. The faster FAD∗ decay is in good agreement with the theoretical expectation that the replacement of the aspartic acid with a neutral or even positively charged amino acid will accelerate the electron transfer process as it alters the active site potential (38).

### Ultrafast transient fluorescence spectroscopy experiments

To gain further information on the impact of D67N on the electron transfer process in OaPAC, we performed ultrafast transient fluorescence measurements on our home-built Kerr-gated fluorescence setup (47). The advantage of using the Kerr-gate approach compared to fluorescence upconversion is that the fluorescence emission spectrum can be obtained at every time delay whereas in the fluorescence upconversion method the decay of the fluorescent intensity is measured at one certain wavelength. Figure 3, *A* and *B*) show the EAS spectra obtained from the global fitting of the transient fluorescence dataset. The maximum of the fluorescence emission is at ∼ 513 nm, which is slightly higher than that observed in AppA BLUF (48) (∼500 nm) and significantly lower than of the free flavin (∼530 nm). The fluorescence emission spectrum indicates that the flavin is embedded in a nonsurface exposed environment. There is no significant difference between the emission maxima of WT and mutant OaPAC, suggesting that the mutation did not result in significant change of the flavin environment.

The fluorescence of WT OaPAC, which was not reported before, was found to be highly dispersive as was observed in the case of AppA (6). The transient fluorescence dataset could be well described with the same three lifetimes (5 ps, 83 ps, and infinite) as retrieved in the TA experiments. The corresponding EAS (species-associated spectra assuming a sequential scheme (1→2→3)) all peaked at ∼ 513 nm, implying that they arise from protein-bound flavin, as the fluorescence emission maximum of free flavin is ∼ 530 nm.

A global fit also resolved three components for the D67N mutant (5 ps, 60 ps, infinite, Fig. 3*B*), again with a shorter second time constants compared to the WT protein. The kinetic traces observed at 513 nm were overlaid (Fig. 3*C*) to compare the decay of the fluorescence emission of WT and D67N. The time constant of the excited state relaxation in D67N is almost half compared to that observed in the WT protein. This assessment, which is not complicated by contributions of product states as in TA experiments, unambiguously demonstrates that overall electron transfer is faster in D67N OaPAC mutant. Yet, as shown above, this modestly faster initial electron transfer does not lead to a higher yield of the signaling state, presumably due to strongly enhanced back PCET from FADH^•^ to the resting dark state (49).

It is worth mentioning that the aspartate to asparagine mutation was suggested for PixD where the authors expected that the mutation would enhance significantly the electron transfer kinetics (38). We performed this homologous mutation in PixD (D69N) and it also resulted in faster fluorescence decay and shorter time constants (Figs. S4 and S5). We also performed TA measurements on PixD WT and D69N and we observed a similar shortening of the time constant of EAS2 (Fig. S6).

### Indirect measurement of cAMP

An enzymatic assay was employed to examine the impact of accelerating the electron transfer process in the D67N mutant blue light sensing domain on the ability of the AC domain to convert ATP to cAMP plus pyrophosphate. The adenylate cyclase activities of 1 μM of WT OaPAC and D67N OaPAC were monitored using a spectrophotometric assay that detects pyrophosphate released by OaPAC when it converts ATP to cAMP. The inorganic pyrophosphatase enzyme converts pyrophosphate into two equivalents of phosphate which is then consumed by the 2-amino-6-mercapto-7-methylpurine riboside/purine nucleoside phosphorylase reaction and detected by an increase in absorbance at 360 nm. Figure 4*A* shows the dark and the light-induced enzymatic activity of WT OaPAC and D67N mutant OaPAC in the presence of 500 μM ATP. In the dark, the enzymatic activity of the mutant is slightly higher than WT but still very low. Upon illumination the conversion rates strongly increased for WT and the D67N mutant. However, D67N mutant converts ATP at a higher rate than WT making this mutant a starting point for tuning PACs as optogenetic tool.

The enzymatic assays were performed using increasing amount of substrate and the results were evaluated using the classical Michaelis–Menten presentation (Fig. 4*B*). The enzymatic assays show an elevated cAMP production in D67N OaPAC: the maximal velocity of the conversion rate was ∼ 1.5 times higher in the mutant (0.100 ± 0.002 mM/min) than in WT (0.064 ± 0.007 mM/min). The concentration of half-maximal velocity (*K*_M_) is also slightly higher in the mutant than in WT —but more importantly the catalytic constant (*k*_cat_)–which gives the number of substrate molecule each enzyme site can convert to product per unit time is ∼ 1.5 times higher in D67N (50.05 1/min) than the WT OaPAC (32.2 1/min) (see Table 1).

**Table 1:** Kinetic parameters for WT OaPAC and D67N mutant

Parameters	WT OaPAC	D67N mutant
*v*_max_	0.064 ± 0.007 mM/min	0.100 ± 0.002 mM/min
*k*_cat_	32.2 1/min	50.05 1/min
*K*_M_	0.031 ± 0.001 mM	0.041 ± 0.005 mM
Dark state recovery	3.6 s	15.3 s
Excited state relaxation	90 ± 30 ps	64 ± 8 ps
Fluorescence lifetime	40 ± 2 ps	25 ± 2 ps
T_m_	68.1 °C	62.1 °C
ΔH	0.078 J/g	0.069 J/g
Kd	2.2 ± 0.4 mM	7.7 ± 1.6 mM

### Recovery experiments

The electronic spectrum of the mutant shows the typical red shift of the S_0_–S_1_ absorption peak (from 442 nm to 455 nm) after blue light irradiation, resembling WT OaPAC and other BLUF domain proteins (Fig. 5*A*). We measured the dark state recovery of WT and D67N OaPAC to connect the photochemistry of the BLUF domain with the functional dynamics of the AC domain. Light to dark recovery of BLUF domains spans from dozens of minutes to a few seconds. In AppA the photocycle is relatively long, with a recovery lifetime of ∼ 25 min (50), in PixD it is substantially shorter (∼26 s) (8, 19) and in OaPAC it is only a couple of seconds (35). The dark state recovery of D67N mutant and WT OaPAC were monitored at 490 nm and the recovery rate of the mutant is ∼5 times lower (15.3 s) than the recovery rate of WT OaPAC (3.6 s) (Fig. 5*B*). This significant change points to a possible structural difference between the mutant and the native protein. The slower recovery of D67N implies that after light excitation the protein spends more time in a structure allowing high-rate cAMP conversion. Altogether, the mutant adopts a structure that is not only more favorable for ATP to cAMP conversion but also slows down the rearrangement to the original structure.

### Differential scanning calorimetry measurements

**Figure 6:**
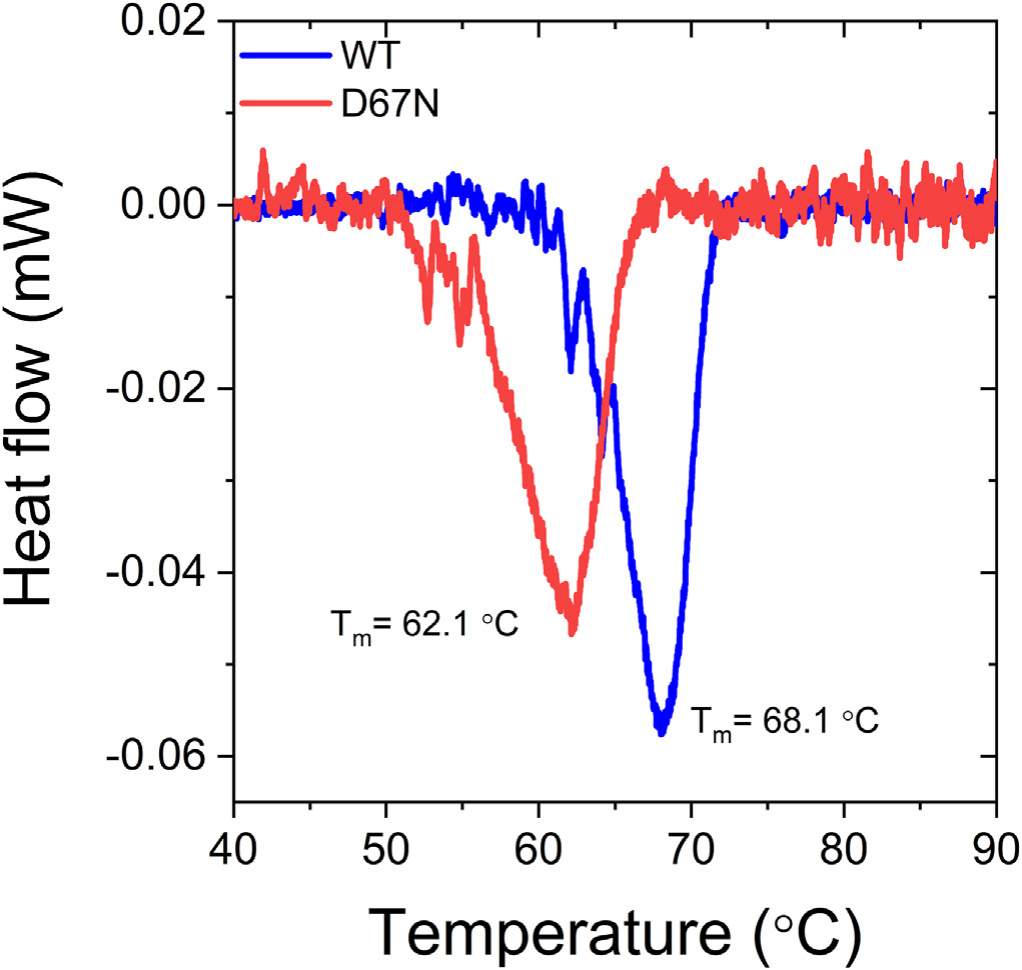
**Thermal unfolding of WT and mutant (D67N) OaPAC measured by DSC.** The DSC curve of the WT shows a steep endothermic unfolding with a melting temperature of 6 °C higher than of the mutant. This points to a more stable or packed structure of the WT protein as denaturing starts at lower temperature in the case of the mutant. DSC, differential scanning calorimetry; OaPAC, photoactivated adenylate cyclase from *Oscillatoria acuminata*.

The difference in the enzymatic activity and the dark state recovery suggests that the introduced mutation has changed the overall protein structure. To test this hypothesis, we performed differential scanning calorimetry (DSC) experiments on both proteins to examine their thermostability. These measurements reveal a significant difference between mutant and WT OaPAC. The thermal denaturation of WT showed a steep endothermic unfolding with a Tm of 68.1 °C and a ΔH of 0.078 J/g. The measurement of D67N resulted in a lower Tm (62.1 °C) with a lower ΔH (0.069 J/g). The melting temperature (Tm) is where 50% of the protein is denatured, while the area under the curve reflects the required energy for protein unfolding associated with the enthalpy change (ΔH). The smaller ΔH of D67N indicates a less compact conformation of the D67N mutant. This is also reflected by the considerably (6 °C) lower Tm value (Fig. 6). This observation suggests that this structural change is at the origin of the elevated activity of D67N OaPAC.

### Fluorescence anisotropy-based nucleotide-binding assays

**Figure 7:**
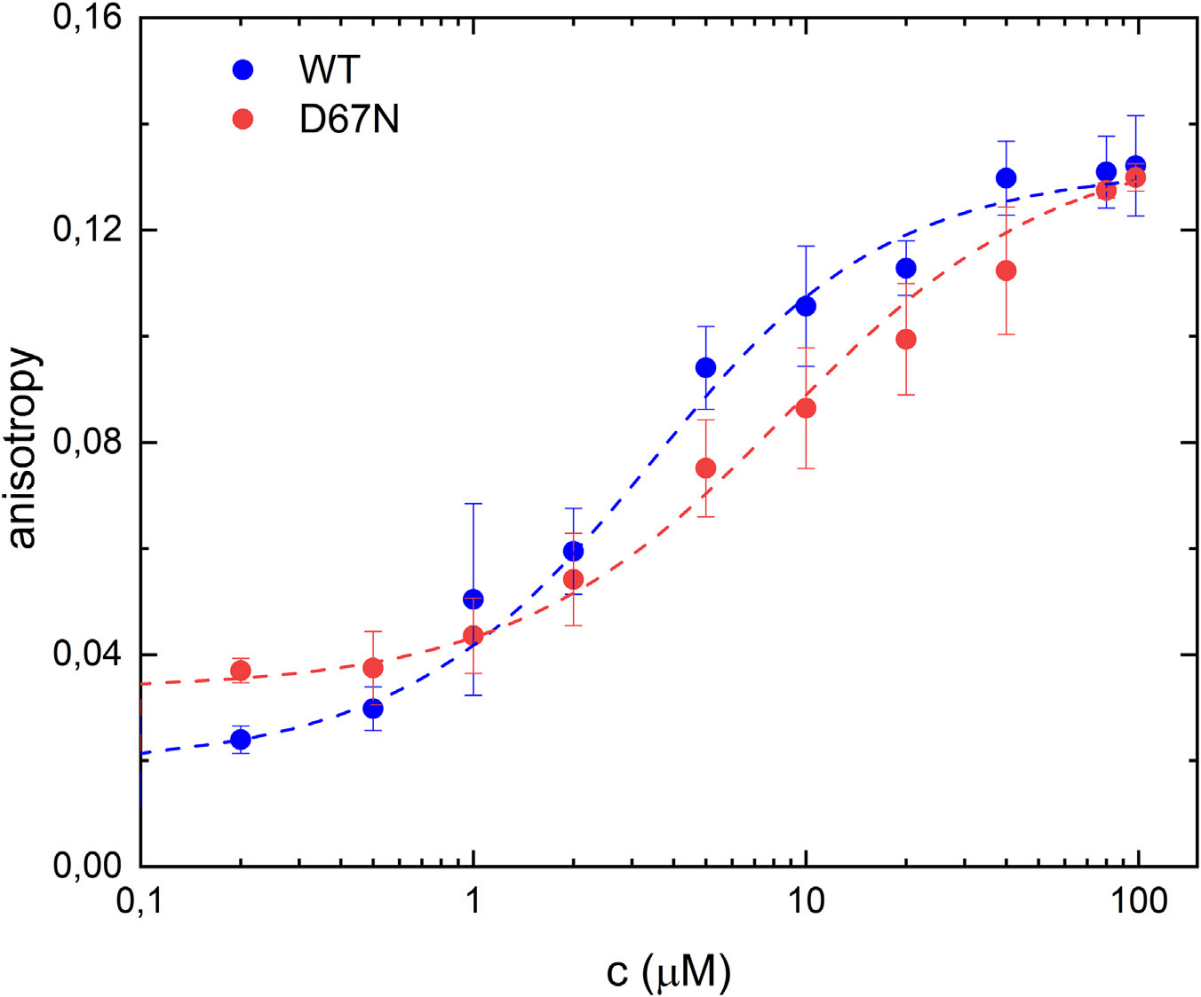
**Fluorescence anisotropy measurement of MANT-ATP nucleotide in WT and D67N OaPAC as a function of protein concentration at fixed (1 μM) MANT-ATP concentration.** As the concentration of protein is increasing, the population of bound nucleotide will also increase resulting in higher fluorescence anisotropy. The obtained binding affinity from the fit was 2.2 ± 0.4 μM in WT and 7.7 ± 1.6 μM in D67N. MANT, 2'-(or-3')-O-(N-methylanthraniloyl) adenosine 5′-triphosphate, trisodium salt; OaPAC, photoactivated adenylate cyclase from *Oscillatoria acuminata*.

It is not known what the affinity of ATP is in the dark- or light-adapted state of the protein. Therefore, using fluorescence anisotropy-based nucleotide-binding assays we measured the binding affinity (*K*_D_) of a fluorescently labeled ATP analog (2′-(or-3′)-O-(N-methylanthraniloyl) adenosine 5′-triphosphate, trisodium salt [MANT ATP]) for the WT and D67N mutant. Fluorescence anisotropy provides a sensitive tool to measure the binding of ligands to proteins when a fluorophore is attached to the ligand (36). Changes in the anisotropy are caused by changes in the mobility of the fluorophore. The addition of OaPAC protein or mutant to MANT-ATP increases the fluorescence anisotropy of the N-methylanthraniloyl (the labeled part of MANT-ATP) as binding of MANT-ATP to OaPAC results in an increase in the volume of the labeled entity and hence slows down its rotational movement. MANT-ATP was excited at λ_exc_ = 350 nm, and the fluorescence anisotropy was detected at 450 nm with increasing concentrations of OaPAC. It should be noted that the flavin in OaPAC emits > 500 nm and therefore there is no contribution from the flavin emission in the anisotropy measurements. The affinity (*K*_D_) for MANT-ATP was determined to be 2.2 ± 0.4 mM for WT and 7.7 ± 1.6 mM for the mutant (Fig. 7). The lower affinity found in the case of the mutant generally aligns with the conclusion that the mutation induces a structural change–as observed in the DSC measurements–which affected the binding affinity of ATP as well as the yield of the cAMP production.

### Concluding remarks

The D67N mutant, which was predicted to change the photocycle kinetics, was made in OaPAC. Based on the crystal structure of OaPAC, D67 is in hydrogen bonding distance to R79 (∼3 Å) residue and to the ribityl chain (∼3.2 Å) of flavin. D67 is a hydrogen bond acceptor and mutating D67 to N, a neutral amino acid, modulates the electrostatic potential of flavin by altering the hydrogen boding network. According to Goings *et al.* (38) the electrostatic potential at the center of flavin can be modulated by either moving positive charge toward the ring or moving negative charge away from the ring. In PixD, the Asp69−Arg71 pair is a good example of residues hydrogen bond to each other that have opposite effects on the active site potential. According to the quantum mechanics/molecular mechanics study done by Goings *et al.* (38), when the Asp69−Arg71 pair moves closer to the flavin ring, the negatively charged Asp69 destabilizes charge transfer, whereas the positively charged Arg71 stabilizes charge transfer, and vice versa. When D67 is mutated to N the hydrogen bond length between the side chain of N67 and the ribityl chain of flavin is expected to increase, potentially favoring charge transfer.

Our ultrafast transient fluorescence measurements demonstrated that the overall kinetics of forward electron transfer was significantly faster in the mutant compared to WT, in qualitative agreement with the predictions of Goings *et al.* (38). The TA measurements also demonstrated ∼4-fold weaker signaling state quantum yield, reflecting a much stronger acceleration of the back reaction competing with formation of the signaling state due to the mutation. A third kinetic effect of the mutation is the ∼5-fold deceleration of the recovery of the dark state. Overall, these counterbalancing effects lead to ∼similar acceleration of the enzymatic rate in WT and D67N OaPAC. However, as the basal activity of D67N OaPAC is somewhat higher than that of WT, the maximal enzymatic velocity *k*_cat_ in the light state is ∼ 1.5 higher in the mutant than in WT. This makes this mutant a better potential optogenetic tool as the basal activities are still low but the light-activated cAMP production is higher than in the native protein.

Upon excitation, the D67N mutant spends longer in the structure favoring the ATP to cAMP conversion. This suggests that the enzymatic activity is increased as the protein spends more in the signaling state despite its lower quantum yield. Our calorimetry measurements showed that the melting temperature is considerably lower than observed in WT, indicating that the D67N mutant unfolds more easily. Hence, the mutant protein seems to possess a less compact structure than WT. The binding experiments also pointed to an altered enzymatic domain structure as WT OaPAC binds ATP slightly more strongly than the mutant.

Overall, future studies aiming at further tuning the relevant kinetic rates may provide a good road to increase the enzymatic activity of PACs.

## Experimental procedures

### Expression and purification of full-length WT and D76N mutant OaPAC

The D67N mutation was generated with Q5 Site-Directed Mutagenesis Kit (NEB) using WT full OaPAC/pCold-I as a template. The mutated construct was verified by DNA sequencing. The full WT or D67N OaPAC/pCold-I construct was transformed into *Escherichia coli* BL21(DE3) cells and grown on an LB-agar plate containing 100 μg/ml ampicillin. A single colony was used to inoculate 10 ml 2×-YT medium (Fisher Bioreagents, BP9743-5) containing 100 μg/ml ampicillin that was shaken overnight at 37 °C (250 RPM). The 10 ml culture was used to inoculate 1 l of 2×-YT medium. The culture was shaken at 37 °C (250 RPM) until the *A*_600_ reached ∼0.8. The temperature was lowered to 18 °C, and following 30 min incubation the protein expression was induced by adding 1 mM IPTG. After 18 h of induction in the dark the cells were harvested by centrifugation and the cell pellet was stored at −20 °C until needed. The cell pellet containing WT or D76N mutant OaPAC was thawed and resuspended in resuspension buffer (50 mM NaH2PO4 pH 8.0, 300 mM NaCl, 2 mg/ml phenylmethylsulphonyl fluoride (PMSF), 1 mg/ml lysozyme, 0.5 mg/ml DNase, Pierce Protease inhibitor tablet (1 tablet/50 ml, Thermo Fisher Scientific)). The resuspended cells were disrupted and lysed by sonication at 4 °C. The cell debris was removed by ultracentrifugation at 30,000 RPM for 80 min at 4 °C. The supernatant was loaded onto a Ni-NTA column equilibrated with resuspension buffer. The column was washed with 60 ml of resuspension buffer containing 5 mM imidazole, and then the protein was eluted using resuspension buffer containing 500 mM imidazole. Buffer content of the eluate was immediately exchanged to 20 mM Tris, 150 mM NaCl pH 8.0, 20 mM MgCl2 using an Econo-Pac 10 G desalting column. The protein was further purified with Superdex 200 column chromatography. Protein purity and yield were determined using SDS-PAGE and UV-visible spectroscopy.

### Picosecond time-resolved fluorescence measurements

Time-resolved fluorescence experiments in the ps time range were performed using a spectrally resolved Kerr-Gate femtosecond fluorometer. The setup employs a Kerr shutter and allows measuring fluorescence spectra with a temporal resolution down to ∼100 fs and up to the nanoseconds timescale. The setup was described elsewhere (47). Briefly, the excitation pulse centered at 390 nm is obtained by frequency-doubling, using a beta barium borate crystal, part of the 780 nm pulse operating at 1 kHz. The remaining 780 nm beam is led through a motorized delay line and focused into the Kerr medium where it spatially overlapped the fluorescence from the sample. The Kerr medium used was CS_2_ (response function width ∼1.2 ps). The sample was flowed through the 1 mm pathlength optical cell using a peristaltic pump. Transient fluorescence spectra were measured with time delays up to 1500 ps for all samples. Global analysis of the time and spectrally resolved data sets in terms of a linear combination of a discrete number of components, each with a distinct exponential rate constant and a decay-associated spectrum (51), was performed using Glotaran (52).

### Ultrafast TA measurements

Transient visible spectra were recorded with 100 fs temporal resolution by a TA spectrometer applying ∼800 μJ laser pulses centered at 800 nm at a repetition rate of 1 kHz. Ultrashort 100 fs pulses were obtained from a Spitfire Ace (Ti:sapphire) regenerative amplifier seeded by a femtosecond Mai Tai mode-locked (Ti:sapphire) laser oscillator and pumped by an Empower 45 multi-kilohertz, intracavity-doubled, green (Nd:YLF) pump laser. The output of the amplifier was split in the ratio 1:9 to build the so-called pump–probe arrangement. The higher energy pulses served to produce, in a BBO crystal, the second harmonic generation 400 nm pump pulses from the 800 nm output of the regenerative amplifier and were attenuated to ∼200 to 400 nJ/pulse before reaching to the sample. The probe arm was provided by the lesser energy laser pulses *via* white continuum generation in a rastered CaF_2_ crystal. The pump and probe pulses were spatially overlapped in the sample and the polarization of the probe was again set to magic angle compared to excitation. To avoid photodegradation, the cuvette was moved with the help of a homemade Lissajous scanner, simultaneously flowed by a peristaltic pump and kept at 12 °C temperature during the whole measurement. A Newport (IMS Series High-Performance Long Travel Linear Stages 600 PP) delay stage was placed in the beam path of the pump pulse to adjust the different pump-probe time delays. Exciting pulses were chopped by a Thorlabs MC2000 optical chopper (to half of the output repetition rate) to generate “pump ON” and “pump OFF” states of the sample. Absorption spectra were recorded by an Andor Newton CCD operating at −80 °C. Absorption data matrices were collected, and the absorption changes calculated, recorded, and stored by the home written NI LabVIEW (https://www.ni.com/hu-hu/shop/labview.html) (visual programming language) data acquisition and control software. The absorption changes were reported as pump on–pump off normalized difference spectra. The obtained data matrix was analyzed by the Glotaran software assuming a sequential scheme with EAS assigned to the obtained time constants.

### cAMP yield measurement/adenylate cyclase activity

The ATP-cAMP conversion of WT and D67N mutant OaPAC was quantified using a pyrophosphate assay (EnzChek Pyrophosphate Assay Kit). This assay is based on the PPi-dependent increase of the absorption of 2-amino-6-mercapto-7-methypurine, which was monitored as a function of time at 360 nm. The reaction rate was determined from the slope of a linear fit using an extinction coefficient of 11,000 M^−1^ cm^−1^ at 360 nm. From the slope of the change of the absorbance the reaction rate (μM/s) of the purine base product (2-amino-6-mercapto-7-methypurine) was determined which is the same as the reaction rate of pyrophosphate derived from ATP.

To determine the Michaelis–Menten constant, the assay was performed on in the presence of 0 to 500 μM concentrations of ATP using the same condition of the continuous illumination. The initial reaction rate at each ATP concentration was extracted from the linear part of *A*_360_ *versus* time plot. The resulting rate constants were plotted as a function of ATP concentration. Fitting a Michaelis–Menten saturation curve for the enzyme reaction, the maximum reaction rate (*V*_max_), the concentration at the half of the maximum *K*_M_ as well as the *k*_cat_—which is the number of ATP molecule each OaPAC converts to cAMP per unit time—were determined.

### DSC measurements

DSC was performed to measure the thermal stability of the WT and D67N mutant OaPAC using a SETARAM Micro DSC-III calorimeter. The measurements were carried out in the range of 20 to 100 °C with a heating rate of 0.3 K·min^−1^. The sample (WT and D67N) and the reference (buffer) were balanced with a precision of ± 0.05 mg in order to avoid corrections for the heat capacity of the vessels. A second thermal scan of the denatured sample was measured for baseline correction. The melting temperature (T_m_) of the thermal unfolding curves were analyzed by the OriginLab Origin2021 software (https://www.originlab.com/).

### Fluorescence anisotropy-based nucleotide binding assays

Fluorescence anisotropy-based nucleotide binding assays were performed at room temperature using 2 μM MANT ATP. This is a hydrolyzable fluorescently labeled ATP, on excitation at 350 nm, emits at ∼450 nm. Steady-state fluorescence anisotropy measurements were performed with a Fluorolog Jobin Yvon Horiba spectrofluorometer in L-format configuration equipped with a polarization accessory. The measurements were performed at an excitation wavelength of λexc = 350 nm with a vertical polarization filter and by measuring the emission at 450 nm (average of 30 measurements on the same sample) with the polarization filter both parallel and perpendicular with respect to the excitation light polarization. Fluorescence anisotropies were calculated from the fluorescence intensities detected according to the Equation 1.

(1) $$r=\frac{I_{perp/perp}-G\left(\lambda \right)I_{perp/par}}{I_{perp/perp}+2G\left(\lambda \right)I_{perp/par}}$$

where r is the fluorescence anisotropy, I_perp/perp_ is the fluorescence emission intensity detected with vertical polarization, I_perp/par_ is the fluorescence emission intensity detected with vertical polarization on the excitation and horizontal polarization on the emission, and G(λ) is the correction factor experimentally determined measuring the ratio I_perp_/I_par_ with a horizontally polarized excitation. Data processing was done using Origin 2020 software (OriginLab, https://www.originlab.com/) and *K*_D_ values were determined by fitting to a quadratic binding equation.

(2) $$\frac{r-r_{A}}{r_{AT}-r_{A}}=\frac{A_{0}+T_{0}+K_{D}-\sqrt{{\left(A_{0}+T_{0}+K_{D}\right)}^{2}-4\cdot A_{0}\cdot T_{0}}}{2}$$

where A_o_ and T_o_ are the total MANT-ATP/cAMP and OaPAC concentrations respectively, r_A_ is the steady-state anisotropy of MANT-ATP/cAMP, r_AT_ is the steady-state anisotropy of MANT-ATP/cAMP at a saturating amount of OaPAC and K_D_ is the dissociation equilibrium constant of the MANT-ATP/cAMP-OaPAC complex.

## Data availability

Data are available in the Supporting information. All remaining data are contained in the article.

## Supporting information

This article contains supporting information (38, 53, 54, 55).

## Conflict of interest

The authors declare that they have no conflicts of interest with the contents of this article.
