# Supplementary material for: Photocycle alteration and increased enzymatic activity in genetically modified photoactivated adenylate cyclase OaPAC
Source: J Biol Chem. 2023 Jul 17;299(8):105056. doi: 10.1016/j.jbc.2023.105056 (PMC10448171; doi:10.1016/j.jbc.2023.105056)
Supplement: Supporting Information [file mmc1.pdf]

1. Spectral modelling of the transient absorption spectra

In order to find the presence of the different radical species we used reference spectra of each plausible radical. The flavin radical spectra were taken from measurements performed on mosquito AgCRY1 (*Anopheles gambiae*). (1, 2) Fig. S1 also contains the spectra of tryptophan radicals determined by pulsed radiolysis experiments.(3) The EAS2 (65 ps) spectra could be fit with a combination of spectra of the flavin excited state and the neutral radical state.

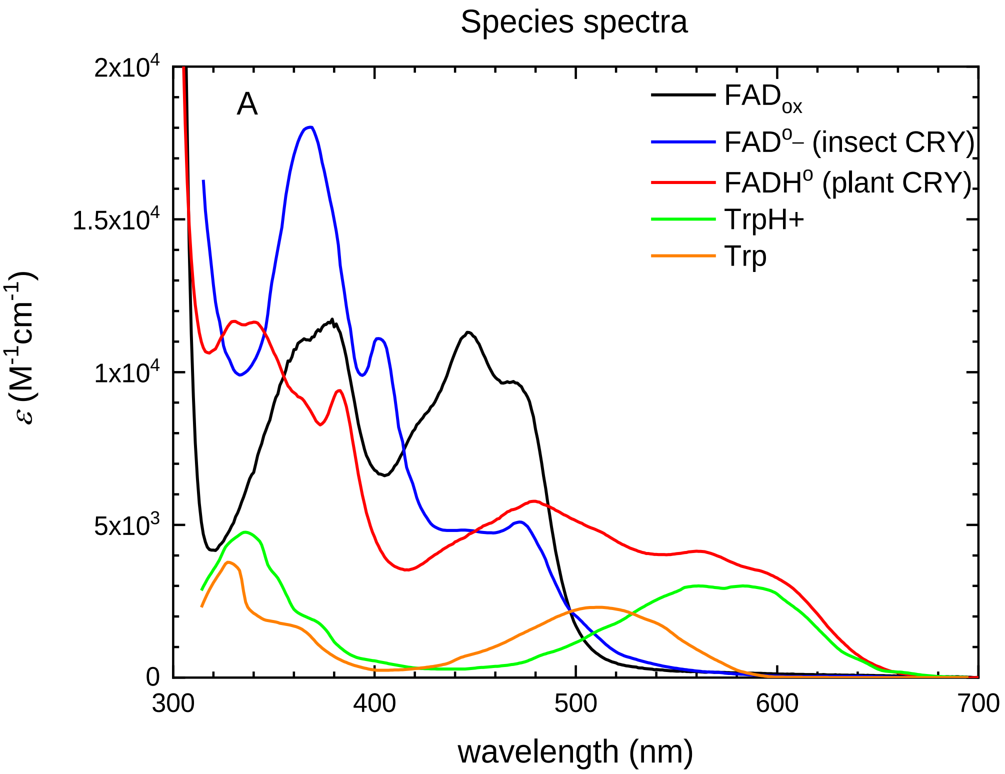

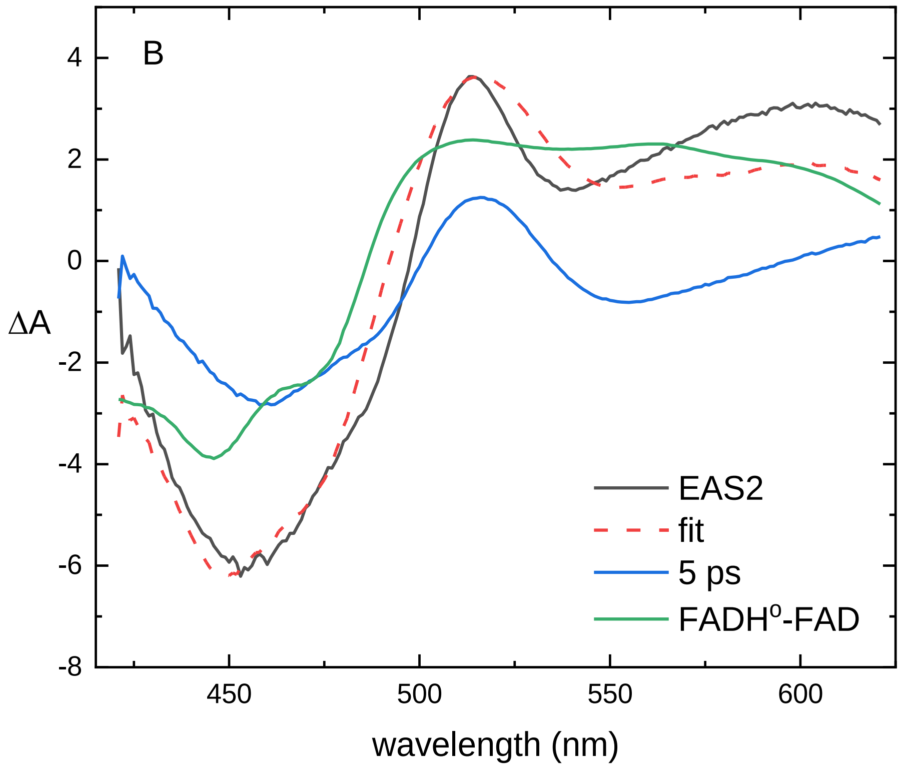

Figure S1. Spectral fitting of **A)** Spectra of the oxidized FAD and the radical FAD species as well as the spectra of the tryptophan radicals. As TyrH and Tyr° do not absorb at >420 nm, their contributions were not taken into account. **B)** 65 ps component for D67N-OaPAC was fitted as a linear combination of (FADH°–FAD) and (FAD*-FAD_{ox}) spectra, as from the fluorescence measurements can be seen that the excited state is present at the longer time constant as well. The FADH° and FAD_{ox} spectra from fig S1A were used to construct the (FADH°–FAD) spectrum and the 5-ps EAS was used as (FAD*-FAD_{ox}) spectrum.

2. PixD WT and D69N fluorescence data

To check the effect of the mutation proposed by Goings et al.(4) in PixD we made the mutant and performed transient fluorescent measurement on WT as well as on the D69N mutant. The fluorescence decay of WT PixD was heterogeneous, similar to WT OaPAC with longer time constants. The time constants of the D69N mutant were very close to the observed in OaPAC D67N mutant. Observing the fluorescence decay of WT and D69N at 520 nm, one can see only a slightly faster initial decay of the fluorescence emission in the mutant.

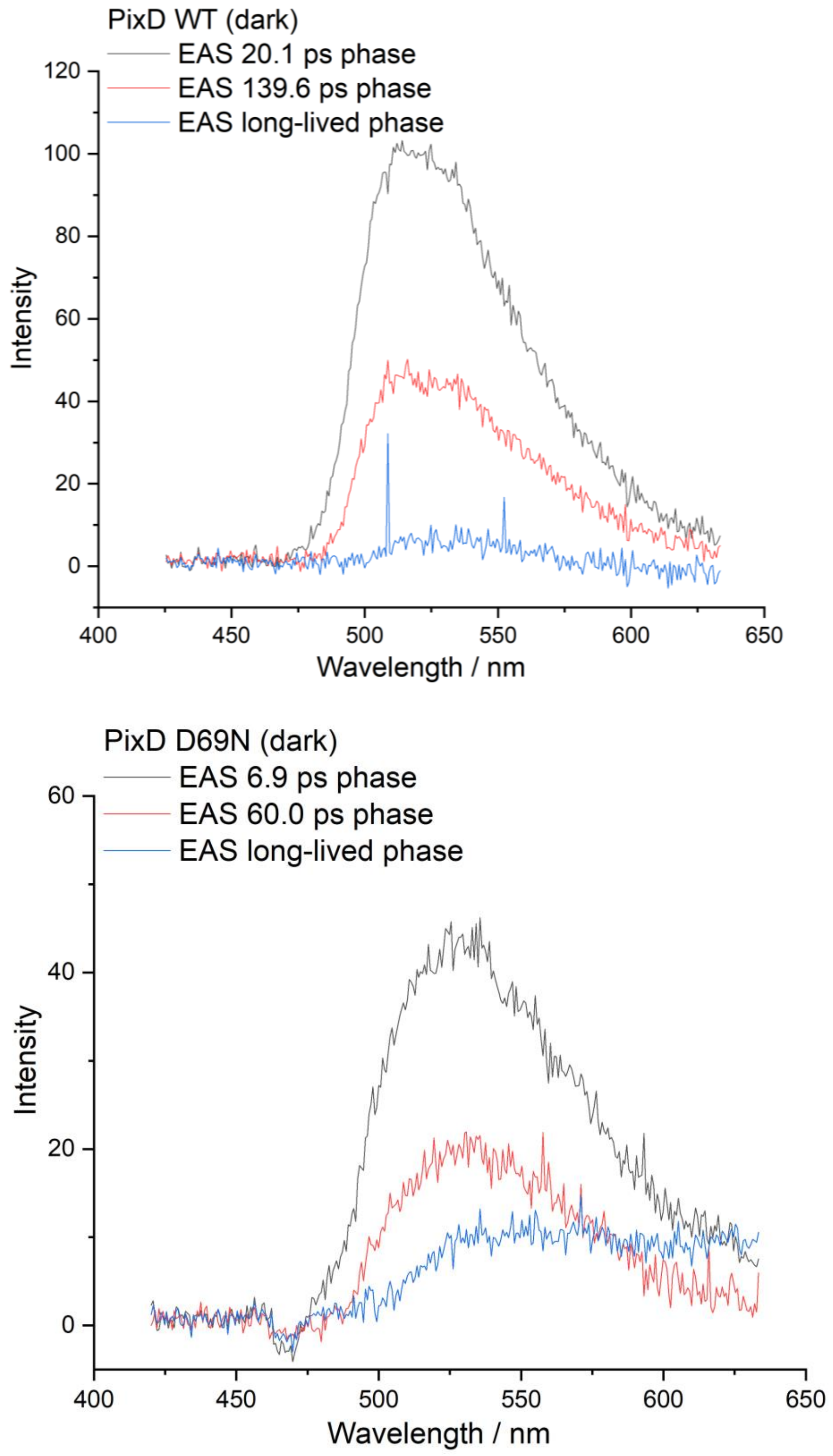

Figure S2. A) EAS spectra obtained in the case of WT B) In D67N mutant.

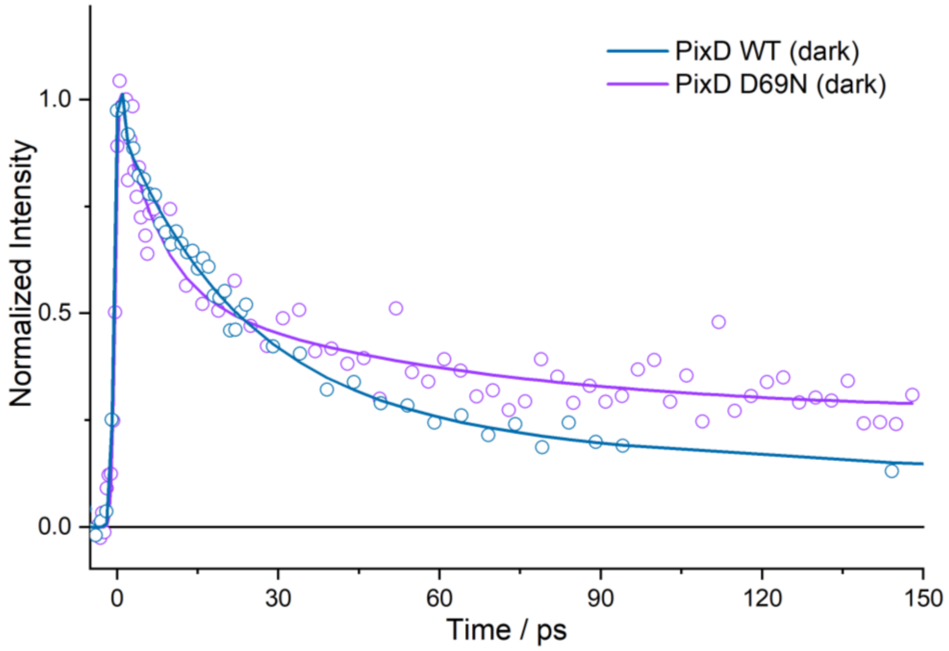

Figure S3. Individual kinetics of WT PixD and D69N PixD observed at 520 nm

3. **Transient absorption measurements on WT and D69N PixD**

We performed transient absorption measurements on WT PixD and on PixD D69N mutant and we observed a rather similar behaviour as seen in OaPAC. The back transfer is faster in the mutant than in WT.

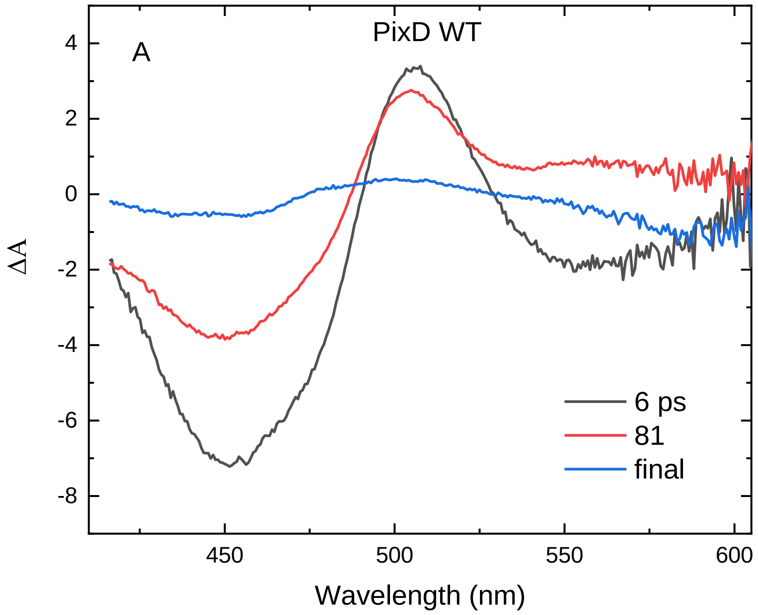

4.

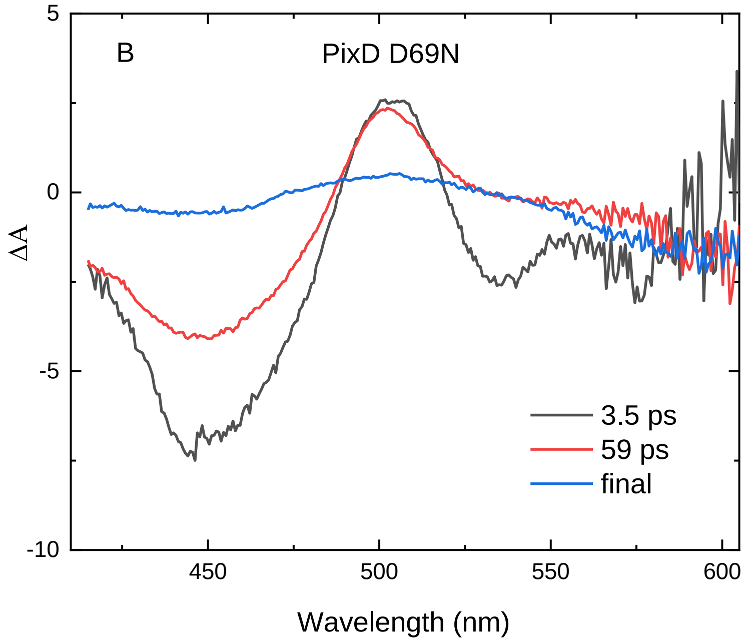

Figure S4. EAS spectra after global analysis on A) WT and B) D69N data

5. Kinetics observed by transient absorption in D67N at 600 nm

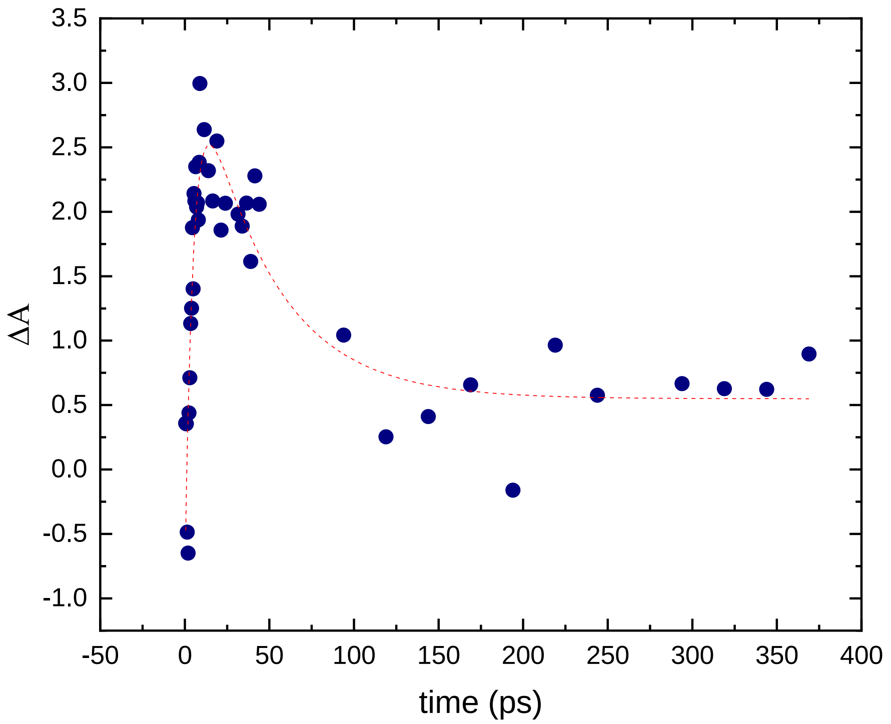

Figure S5. Kinetics observed at 600 nm where formation of FADH• should appear. The formation of the neutral radical state and happens with a time constant of 5.1 ± 1.5 ps, relaxation (deprotonation) of this state happens with a time constant 42.5 ± 18 ps. The two time constants overlap with the values of EAS1 and EAS2 determined by the global fit.

6. **Not normalized EAS spectra of WT and D67N OaPAC**

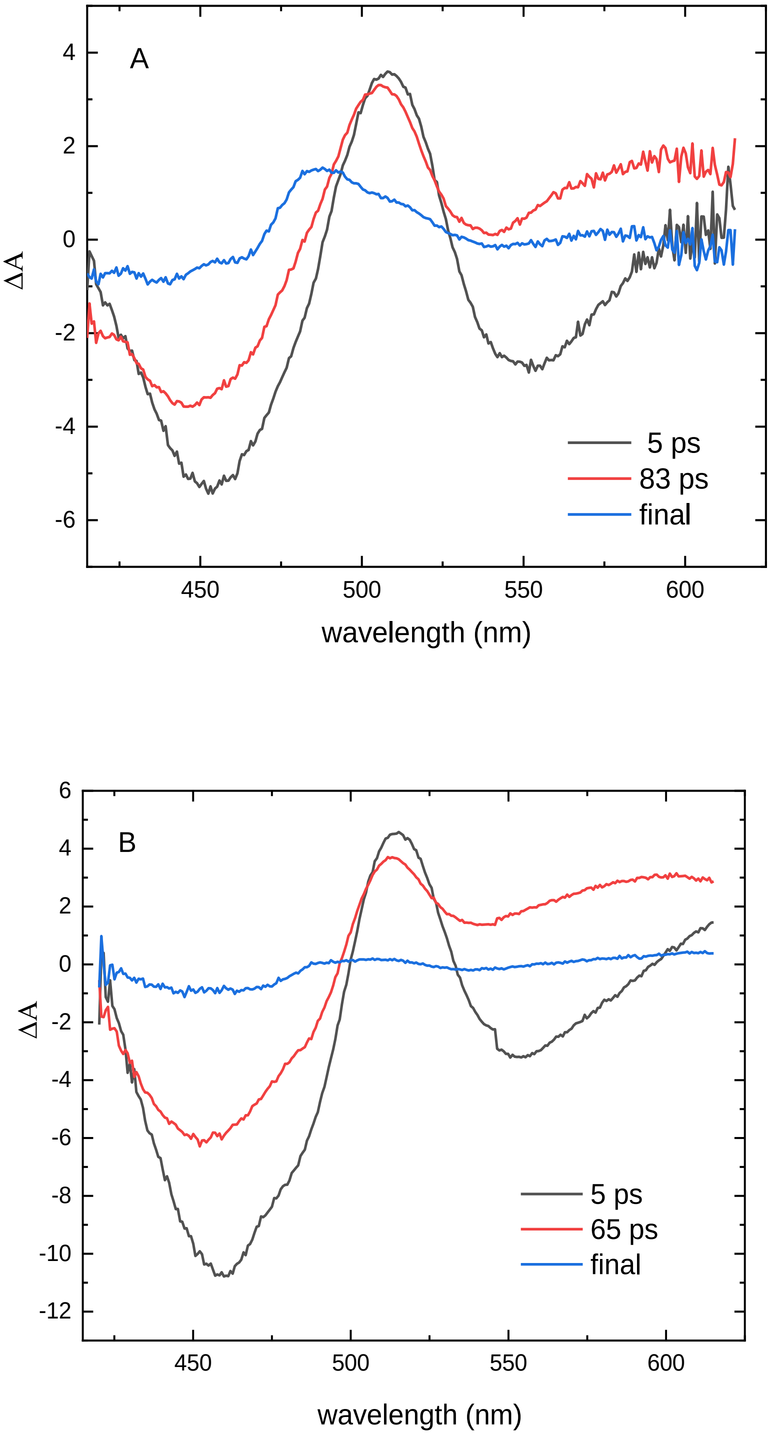

Figure S6. Not normalized EAS spectra after global analysis on WT and D67N data. The amplitude of the final state is less than 10 % of the first EAS in the case of D67N measurement

References

1. Liu, B., Liu, H., Zhong, D., andLin, C. (2010) Searching for a photocycle of the cryptochrome photoreceptors Curr Opin Plant Biol **13**, 578-586 10.1016/j.pbi.2010.09.005
2. Liu, Z., Zhang, M., Guo, X., Tan, C., Li, J., Wang, L. *et al.* (2013) Dynamic determination of the functional state in photolyase and the implication for cryptochrome Proceedings of the National Academy of Sciences of the United States of America **110**, 12972-12977 10.1073/pnas.1311077110
3. Solar, S., Gtoff, N., Surdhar, P. S., Armstrong, D. A., andSingh, A. (1991) Oxidation of tryptophan and N-methylindole by N3•, Br2•- and (SCN)2•- radicals in light- and heavy-water solutions: a pulse radiolysis study Journal of Physical Chemistry 3639-3643
4. Goings, J., andHammes-Schiffer, S. (2019) Early Photocycle of Slr1694 Blue-Light Using Flavin Photoreceptor Unraveled through Adiabatic Excited-State Quantum Mechanical/Molecular Mechanical Dynamics Journal of the American Chemical Society **141**, 20470-20479 10.1021/jacs.9b11196
